# Single- and Multilayer Build-Up of an Antibacterial Temperature- and UV-Curing Sol–Gel System with Atmospheric Pressure Plasma

**DOI:** 10.3390/gels9090675

**Published:** 2023-08-22

**Authors:** Simon Chwatal, Sabine Pölzl, Clemens Kittinger, Jürgen Markus Lackner, Anna Maria Coclite, Wolfgang Waldhauser

**Affiliations:** 1 Joanneum Research Forschungsgesellschaft mbH, MATERIALS-Institut für Oberflächentechnologien und Photonik, Leobner Strasse 94a, 8712 Niklasdorf, Austria; juergen.lackner@joanneum.at (J.M.L.); wolfgang.waldhauser@joanneum.at (W.W.); 2 Diagnostic & Research Institute for Hygiene, Microbiology and Environmental Medicine, Medical University of Graz, Neue Stiftingtalstrasse 6/III, 8010 Graz, Austria; sabine.poelzl@medunigraz.at (S.P.); clemens.kittinger@medunigraz.at (C.K.); 3 Institute for Solid State Physics, Graz University of Technology, Petersgasse 16/III, 8010 Graz, Austria; anna.coclite@tugraz.at

**Keywords:** sol–gel coatings, atmospheric pressure plasma, surface functionalization, multilayer coating, antibacterial, scratch resistance

## Abstract

The versatility of sol–gel systems makes them ideal for functional coatings in industry. However, existing coatings are either too thin or take too long to cure. To address these issues, this paper proposes using an atmospheric pressure plasma source to fully cure and functionalize thicker sol–gel coatings in a single step. The study explores coating various substrates with sol–gel layers to make them scratch-resistant, antibacterial, and antiadhesive. Microparticles like copper, zinc, or copper flakes are added to achieve antibacterial effects. The sol–gel system can be sprayed on and quickly functionalized on the substrate. The study focuses on introducing and anchoring particles in the sol–gel layer to achieve an excellent antibacterial effect by changing the penetration depth. Overall, this method offers a more efficient and effective approach to sol–gel coatings for industrial applications. In order to achieve a layer thickness of more than 100 µm, the second part of the study proposes a multilayer system comprising 15 to 30 µm thick monolayers that can be modified by introducing fillers (such as TiO_2_) or scratch-resistant chemicals like titanium isopropoxide. This system also allows for individual plasma functionalization of each sol–gel layer. For instance, the top layer can be introduced with antibacterial particles, while another layer can be enhanced with fillers to increase wear resistance. The study reveals the varying antibacterial effects of spherical particles versus flat flakes and the different scratch hardnesses induced by changes in pH, number of layers, and particle introduction.

## 1. Introduction

Historically, the scope of sol–gel coating research has been quite narrow. To achieve optimal results with applied sol–gel layers, it is important to consider the thickness of the coating and the curing process. For thinner layers, a quick cure using an oven or UV light is sufficient [1]. However, thicker layers require a longer curing time in the oven, typically several hours, to ensure all possible reactions in the coating are completed [2]. The combination of both with a strong emphasis on short bursts of high-energy input has not been thoroughly explored. Few approaches to solving this curing problem are addressed in the literature [3,4,5,6]. Similarly, the functionalization of the coatings during the curing process is rarely described.

By utilizing an atmospheric pressure plasma setup, it is possible to expedite the curing process for various sol–gel systems on a range of substrates, including stainless steel, plastics, and glass. This innovative approach offers a fast and efficient means of curing select UV-sol–gel systems, making it an ideal option for producing thin functional films for diverse applications. With its exceptional potential for economic realization, this technology holds great promise for enhancing the efficiency and speed of curing processes.

Another advantage is the possibility to further modify and functionalize the layers during the curing process. Such functionalizations significantly change the properties of the sol–gel layer. It is possible to achieve an antibacterial effect by adding copper or zinc particles or copper flakes to the layer [7,8], a better scratch resistance, or a curing effect by introducing different particles.

By combining the sol–gel with precursors, e.g., hexamethyldisiloxane (HMDSO), the surface energy of the layer can be changed. It is thus possible to apply a hydrophilic HMDSO layer to a hydrophobic sol–gel layer. This change makes it possible to achieve a self-cleaning effect due to the high wettability.

As Yang et al. [9] described, adding titanium isopropoxide (TTIP) can improve silicon–oxygen–carbon-based coatings’ temperature resistance and hardness. Since the sol–gel used in this work mainly comprises these three elements, experiments were undertaken with TTIP, which should show an improvement in scratch resistance.

This paper continues the work started by Chwatal et al. [10]. The first paper incorporated copper particles into a sol–gel monolayer to explore their antibacterial and antiviral effects and developed a suitable sol–gel system for plasma curing. However, a post-treatment step was required to expose the particles and enhance the coating’s antimicrobial activity. In this study, we aimed to eliminate the need for a post-treatment step by depositing the particles directly onto the surface through adjustments in plasma parameters. Zinc was also included in the study, and copper flakes were used as an alternative to spherical particles. The flakes were chosen for their larger surface area, which resulted in a higher concentration of copper on the surface, and their shape, which prevented deep penetration into the layer.

This paper comprehensively analyses the differences between monolayer and multilayer systems in terms of hardness and curing [11,12]. Monolayer systems involve applying a single sol–gel layer to the substrate and curing it. In contrast, multilayer systems require applying an adhesion promoter layer after the first sol–gel layer has cured, followed by alternating layers of sol–gel and adhesion promoter until the desired thickness is achieved. The study reveals a significant difference in scratch hardness between the two systems, with the bottom layer of the multilayer setup being softer than the top layers. Due to the different scratch hardness, there are also significant differences in the coefficient of friction (COF). In order to alter the properties of these systems, functionalization with particles and precursors is utilized. This functionalization is completed during the sol–gel production by adding TTIP, TiO_2_ powder, or HCl/NaOH, and later, by feeding powder (Cu and Zn) or a precursor (HMDSO) into the plasma during curing. The results of these experiments are briefly described and analyzed in conjunction with previously published findings and novel discoveries.

## 2. Results and Discussion

### 2.1. Chemical Composition

#### FTIR Studies

**Figure 1:**
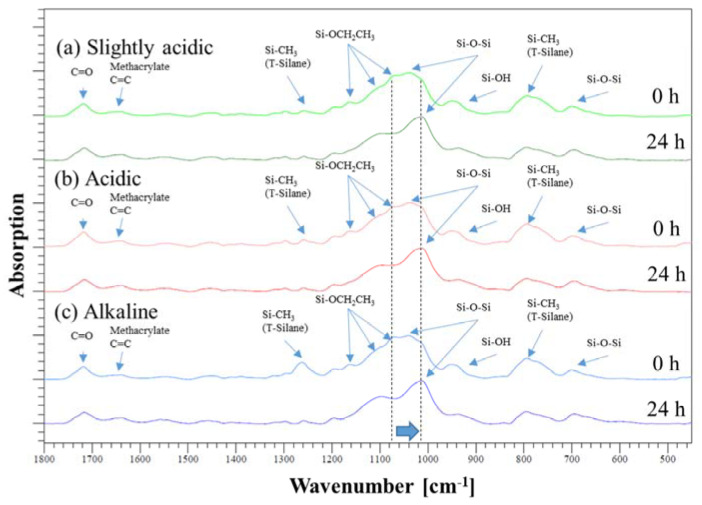
Superimposed spectra of the (**a**) slightly acidic sol–gel (pH ~ 5), (**b**) the acidic sol–gel (pH ~ 3), and (**c**) the alkaline sol–gel (pH ~ 13). Out of the measured states, only two are currently displayed. One measurement was taken immediately after coating and curing, specifically at 0 h and 24 h after the curing. The samples were stored under normal room conditions, without exposure to UV or heat.

FTIR spectroscopy allows determining the reaction progress and the degree of poly-condensation in the layer at different times. Therefore, it is possible to describe chemical reactions and show their effects on the curing behavior. The primary reaction is described by the polycondensation of the Si-ethoxy groups (1067 cm^−1^) among themselves to form a Si-O-Si network (1013 cm^−1^). A shift from the peak at 1067 cm^−1^ to the Si-O-Si peak at 1039 cm^−1^ and finally to the Si-O-Si peak at 1013 cm^−1^ can be seen (Figure 1, Table 1). Out of the measured states, only two are currently displayed. One measurement was taken immediately after coating and curing, specifically at 0 h and 24 h after the curing. The samples were stored under normal room conditions, without exposure to UV or heat. Out of the measured states, only two are currently displayed. One measurement was taken immediately after coating and curing, specifically at 0 h and 24 h after the curing. The samples were stored under normal room conditions, without exposure to UV or heat. The absence of any discernible differences between the individual spectra, except for this shift, indicates that the chemical reaction that occurs is limited to polycondensation.

The degree of cross-linking was estimated as the ratio between the two prominent peaks at 1067 cm^−1^ and 1013 cm^−1^. The higher this value, the more completely the reaction progressed.

**Figure 2:**
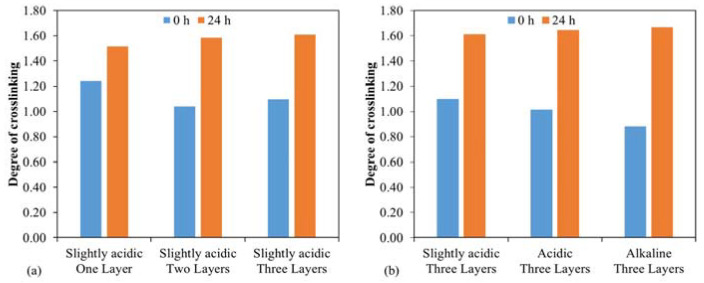
(**a**) Comparison of the degree of cross-linking based on the applied and cured layers (one, two, and three sol–gel layers); (**b**) Comparison of the degree of cross-linking based on the pH in the applied sol–gel system (Slightly acidic pH ~ 5, Acidic pH ~ 3, Alkaline pH ~ 13).

In Figure 2a, a monolayer system is more cross-linked immediately after curing (0 h) than the multilayer systems. The post-cure phase (24 h) completely compensates for this difference, so all three systems are very well cured to the same extent. The post-curing process in multilayer coatings primarily occurs in the top layer, as evidenced by FTIR measurements that exclusively evaluate the uppermost micrometers. This finding is further substantiated by scratch hardness measurements, which demonstrate that the scratch hardness of the monolayers is significantly higher than that of the multilayers.

Figure 2b also shows slight differences directly after the plasma treatment step. The sol–gel systems catalyzed at low pH were cured better than the primary system. This difference is no longer present after 24 h.

### 2.2. Film Structure and Surface Properties

#### 2.2.1. Morphology Studies

**Figure 3:**
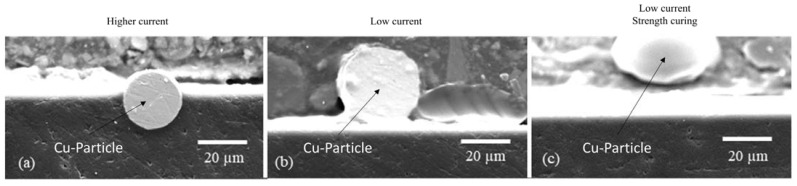
SEM images showing the penetration depth of copper particles depending on the plasma parameters: (**a**) high penetration depth due to high current (180 A) and not yet cured sol–gel layer Cu1 (Table 2 Powder 1, Table 3 Plasma 1); (**b**) low penetration depth due to low current (120 A) Cu2 (Table 2 Powder 2, Table 3 Plasma 1); (**c**) no penetration of the particle into the layer due to low current (120 A) and already slightly precured sol–gel layer (Table 2 Powder 2, Table 3 Plasma 3).

Chwatal et al. [10] emphasized the importance of functionalizing particles and understanding the relationship between plasma parameters and particle penetration depth in monolayer systems. By doing so, the need for post-treatment steps can be avoided while achieving effective antibacterial and antiviral activity. In order to examine the depth of particle penetration into the sol–gel layer, a cross-sectional analysis of the samples was conducted. This process entailed the cutting, embedding, and polishing of the samples. An examination of light microscope and SEM images of variously treated samples showed a significant association between the plasma curing parameters and particle penetration depth. More specifically, a higher current resulted in a greater particle velocity and deeper penetration into the sol–gel layer (Figure 3a). If the current was lowered, the penetration depth also decreased, and the particles lie more or less exclusively on the surface of the sol–gel coating (Figure 3b).

A similar outcome was observed when the sol–gel layer was excessively cured prior to particle application. The coating became well-cured and mechanically rigid, hindering the particles from infiltrating the system (Figure 3c).

To achieve an effective antibacterial effect, the experiments tested various parameters with copper and zinc particles by varying their penetration depth. It was crucial to ensure that the particles did not penetrate too deeply into the layer and instead remained on the surface.

**Figure 4:**
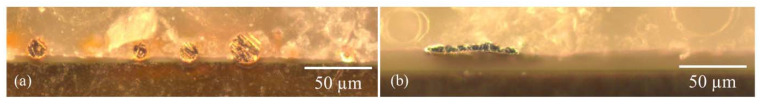
Light microscope images of particle-modified sol–gel layer: (**a**) cross-section light microscope image of the sol–gel layer with copper particles (Cu2) (Table 2 Powder 2, Table 3 Plasma 1); (**b**) cross-section light microscope image of the sol–gel layer with zinc particles (Zn2) (Table 2 Powder 4, Table 3 Plasma 1).

The optical micrographs show the deposition of the particles onto the surface of the sol–gel layer at all three copper and zinc parameters. The plasma did not melt the copper particles; however, the zinc particles were melted (Figure 4).

**Figure 5:**
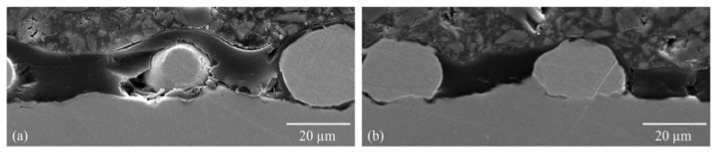
SEM images of the particle-modified sol–gel layer: (**a**) cross-section SEM image of a sol–gel layer with copper particles (Cu4), where the particles are entirely embedded in the layer (Table 2 Powder 1, Table 3 Plasma 4); (**b**) cross-section SEM image of a sol–gel layer with copper particles, where the particles are again exposed on the surface by post-polishing (Cu4 polished) (Table 2 Powder 1, Table 3 Plasma 4).

**Figure 6:**
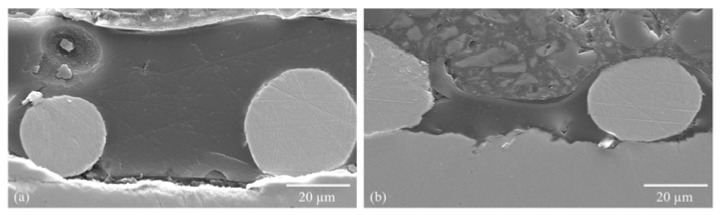
SEM images of the particle-modified sol–gel layer: (**a**) cross-section SEM image of a sol–gel layer with copper particles (Cu5), where the particles are entirely embedded in the layer (Table 2 Powder 1, Table 3 Plasma 2); (**b**) cross-section SEM image of a sol–gel layer with copper particles, where the particles are again exposed on the surface by post-polishing (Cu5 polished) (Table 2 Powder 1, Table 3 Plasma 2).

For better resolution and additional compositional information, the individual sol–gel layers or multilayer systems analyses were analyzed by SEM and EDXS. The SEM images show even more detailed surface structures. EDXS analysis indicated the particle, precursor, and sol–gel distribution (Figure 5 and Figure 6).

As shown later, antibacterial results were promising for all layered-powder systems. The major drawback was the lack of resistance in these biological tests. Accordingly, the particles detached from the layer during or shortly after the tests. This behavior suggests that the particles were not anchored well enough.

Therefore, further tests were carried out. In these experiments, the zinc powder was dispensed with only copper.

Although the powder application and curing process successfully anchored particles into the coating, the sol–gel completely enclosed the powder, resulting in no antibacterial effect. However, the release of particles could be achieved through re-polishing the surfaces. This step restored the desired antibacterial effect without causing particle detachment during biological tests.

**Figure 7:**
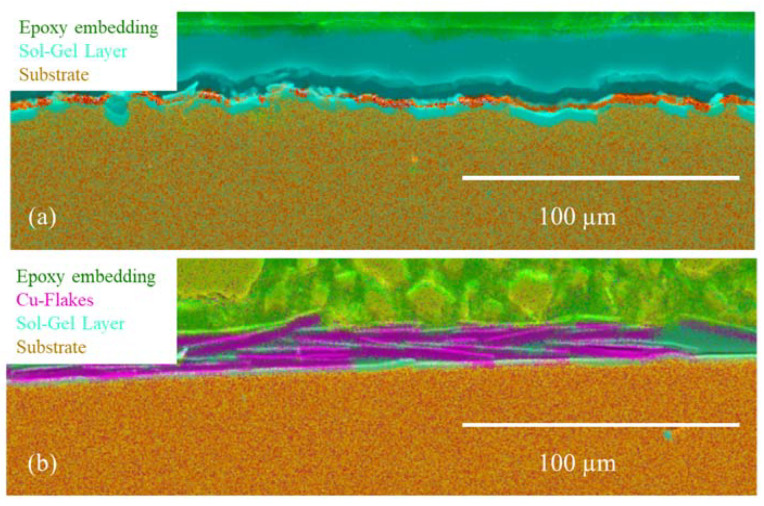
(**a**) Sol–Gel layer without Cu-Flakes; (**b**) Sol–Gel layer with Cu-Flakes (Cu-Flakes 3).

In addition to the “spherical” Cu and Zn particles, Cu flakes were also used. Due to their shape (elongated and flat) and size (approx. 34 µm), these should not sink into the sol–gel and thus be curable at the surface of the sol–gel (Figure 7).

**Figure 8:**
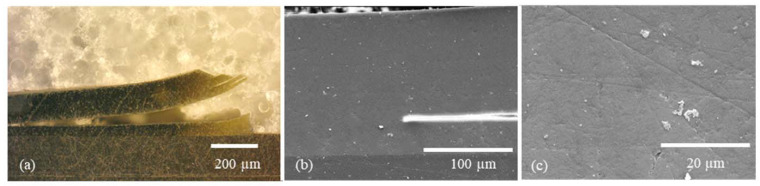
Multilayer structure of a sol–gel system with HMDSO interlayer as adhesion promoter and TTIP mixed in. (**a**) Cross-section optical microscope image at the layer fracture edge; (**b**) cross-section SEM image at the layer fracture edge; (**c**) cross-section SEM image in the layer.

**Figure 9:**
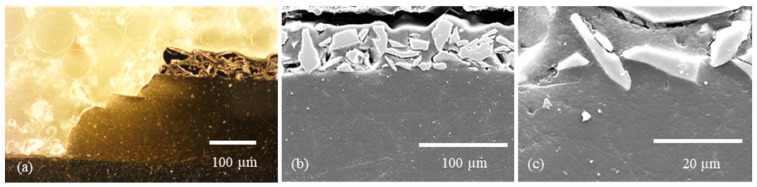
Multilayer structure of a sol–gel system with HMDSO interlayer as an adhesion promoter, TTIP, and TiO_2_ powder mixed in. (**a**) Cross-section optical microscope image at the fracture edge; (**b**) cross-section SEM image at the fracture edge; (**c**) SEM image in the layer.

Light microscope and SEM images were also taken for the more detailed studies of mono- and multilayer coatings subsequently prepared. Among other things, this allows the position of the Cu flakes in the layer to be seen very clearly (Figure 7). The flakes were arranged in a stacked fashion, enabling them to attain the surface of the layer. The intended outcome was attained even though the flakes still penetrated the layer. This surface structure has been shown to be efficacious in the antibacterial assays, which were subsequently performed. The layer-by-layer structure of the sol–gel system was meticulously characterized through a combination of light microscope and SEM analyses (Figure 8a and Figure 9a). In order to prepare cross-sections for analysis, the samples underwent a process of cutting, embedding, and polishing, similar to that employed for monolayer systems. During the cutting process, it was observed that the lowest sol–gel layer detached from the others, thereby demonstrating the strong adhesion of the layer to the substrate. This phenomenon is depicted in Figure 8a and Figure 9a, which illustrate the individual sol–gel layers with HMDSO adhesion layers in between. The top layer was found to contain TiO_2_ particles, which were added as a filler in order to highlight the difference from the other layers and explore the relationship between coating thickness and particle addition. Scanning electron microscope (SEM) images confirmed the compactness of the individual layers and the overall structure of the layer system.

**Figure 10:**
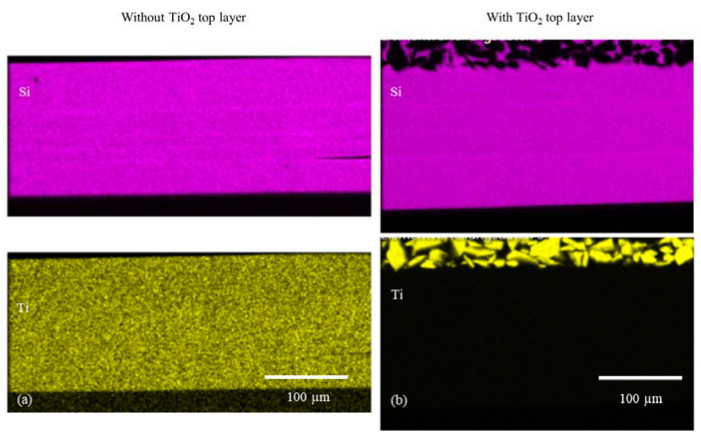
EDXS images of the cured sol–gel layers: (**a**) silicon and titanium distribution in the sol–gel layer (titanium originates from TTIP only); (**b**) silicon and titanium distribution in the sol–gel layer (titanium originates from both TTIP and TiO_2_ powder in top layer).

With the help of the EDXS analysis, element distribution maps were prepared. Contaminations from the sample preparation, such as iron or aluminum, were excluded from the analysis. Although a significant element in the coating, carbon was not considered due to high analytical errors in carbon EDXS analysis. The silicon-rich area represents the sol–gel layer. High titanium contents reflect the distribution of TTIP mixed into the sol–gel and the TiO_2_ introduced into the top layer (Figure 10). It can be seen in Table 4 that the titanium concentration is constant throughout the entire layer. By introducing TiO_2_ particles, the concentration in the surface layer increases significantly.

In contrast to the monolayer systems (15–30 µm) (Figure 3), the alternating sol–gel and hydrophilic HMDSO in the multilayer system allows a film thickness of about 146 µm. Optimizing the parameters and adding TiO_2_ particles could still increase the film thickness to over 230 µm with the same number of layers (Table 5).

As shown in the studies of the monolayer systems, antibacterial properties can be obtained by incorporating copper or zinc particles into the sol–gel layer. If the sol–gel layer is also damaged, no antibacterial or antiviral effect can be guaranteed.

To ensure that the layer still preserves its antibacterial effect even if it is damaged, a kind of reservoir can be created by a multilayer structure. For this purpose, either constantly the same or different particles were introduced during the curing process of a sol–gel layer. The plasma was then applied to the adhesion promoter layer. The process was then repeated. This way, a multilayer structure with particles in each sol–gel layer can be generated.

**Figure 11:**
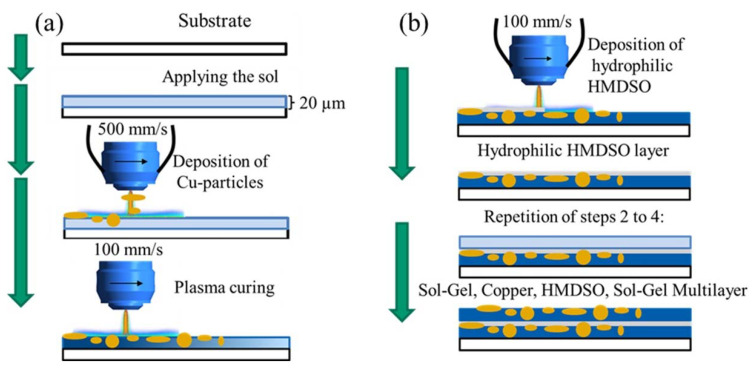
(**a**) Coating process in several steps: sol–gel application manually, functionalization by particles injected into the plasma, curing of the layer by the plasma; (**b**) For multilayer structure: Deposition of a hydrophilic adhesion promoter layer onto a cured sol–gel layer, followed by the repetition of the coating process.

In Figure 11a multilayer setup is displayed. The first sol–gel layer with copper particles was applied to the stainless-steel substrate. On top of this, a hydrophilic HMDSO layer ensures adhesion to the other layers (Table 6). A cured sol–gel layer with copper forms the top layer (Figure 11).

The multilayer system serves the purpose of preventing particles from sinking into deeper layers through curing them in a previous step. This ensures that the underlying layers maintain the necessary antibacterial and antiviral effects in the event of any damage caused to the top layer. The system achieves this by incorporating numerous particles in multiple layers, thereby preventing agglomeration in later-applied layers and resulting in a more uniformly distributed particle distribution.

**Figure 12:**
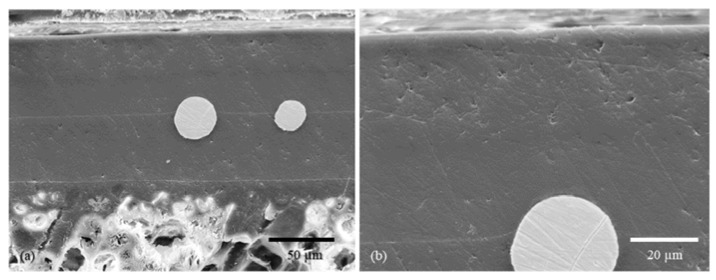
A multilayer sol–gel coating with copper particles integrated through the plasma is shown here. The particles were introduced into the first sol–gel layer and, in a further step, wholly enclosed with sol–gel, including TTIP, to form a reservoir. Figure 14 shows EDXS images of this layer.

**Figure 13:**
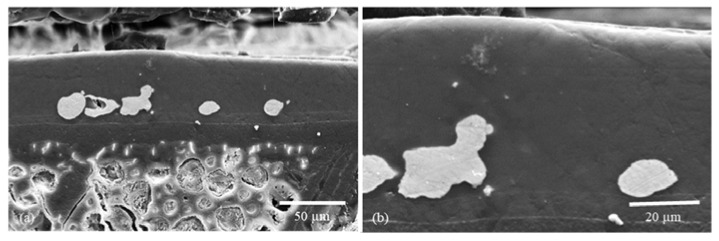
Here, a multilayer sol–gel coating with zinc particles integrated through the plasma is shown. The particles were introduced into the first sol–gel layer and, in a further step, wholly enclosed with sol–gel, including TTIP, to form a reservoir. Figure 15 shows EDXS images of this layer.

In addition to copper, zinc particles were introduced into the layer via plasma. The significant difference in the melting point of these two metals leads to more vigorous melting or deformation of the zinc compared to the copper particles (Figure 12a,b and Figure 13a,b). However, the molten zinc particles are distributed over an even larger area compared to copper and are anchored into each other.

In addition, this process made it possible to apply and cure sol–gels with different chemical compositions on top of each other. For example, TTIP was mixed into a sol–gel to introduce titanium into the layer. Titanium should serve as a starting material for a self-regenerating effect.

**Figure 14:**
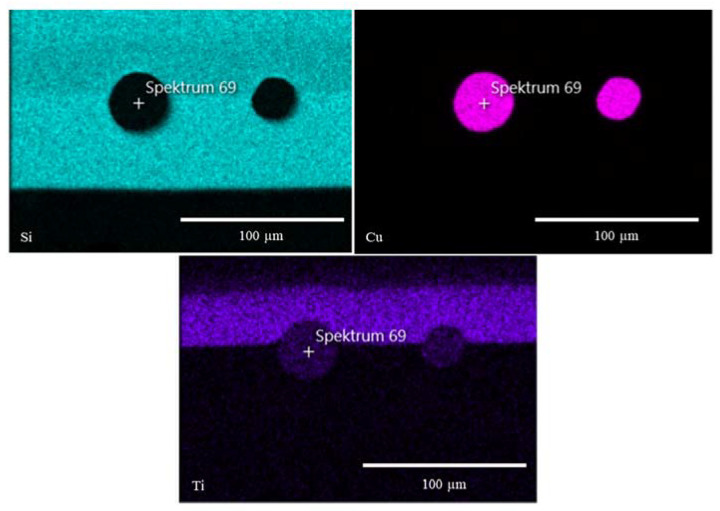
EDXS images show the distribution of silicon, copper, and titanium in the sol–gel system. The copper was introduced into the first sol–gel layer (without TTIP). To generate additional properties, e.g., self-cleaning, TTIP was mixed into the second layer.

**Figure 15:**
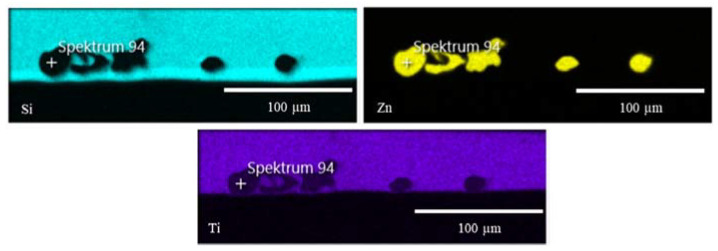
EDXS images show the distribution of silicon, zinc, and titanium in the sol–gel system. The copper was introduced into the first sol–gel layer (without TTIP). To generate additional properties, TTIP was mixed into the second layer.

In the EDXS images Figure 14 and Figure 15, the distribution of the titanium in the layer can be shown very well. Titanium in the form of TiO_2_ or TTIP opens different ways for functionalization and combination of several systems.

#### 2.2.2. Critical Loads and Tribology

In agreement with the findings of previous work [19,20,21], the critical load tests were completed manually to obtain more precise and expressive results. The aim was to improve the already published scratch hardnesses in Chwatal et al. [10] by varying the pH and adding TTIP. The tests with different pH values in the sol–gel systems showed that the monolayers with low pH showed higher scratch resistance (Table 7). As also shown in the work of Gabrielli et al. [22], silanols form more readily at low pH values, which react with each other via condensation. This reaction behavior results in better cross-linking. The same trend can be seen with the multilayers. In these systems, however, the scratch hardness decreases significantly (approx. halved). The incomplete curing process can be inferred from these findings. The lower scratch hardness of the multilayer systems is due to the better curing of the top layers than the first layers, which are applied layer by layer. The FTIR measurements revealed that the multilayer coating, subsequent to plasma treatment, does not exhibit the same level of cross-linking as the monolayer coating. Nonetheless, this effect is compensated for with a 24-h post-curing period in air. It is important to note that FTIR measurements only provide a mapping of the outermost micrometer of the layer rather than the entire layer. Despite this limitation, the scratch hardness of the multilayer coatings is significantly lower than that of the monolayer, which implies that the top layer has undergone curing, while the underlying layers were unable to undergo post-curing. Nonetheless, the addition of TTIP can counter this effect and potentially enhance the scratch hardness of the multilayers.

Plasma parameter set plasma 2 (Table 3) significantly increased the hardness of sol–gel with TTIP multilayer systems, surpassing that of monolayer systems. TTIP plays an essential role in this. As described in the work of Yang et al. [9], TTIP increases temperature resistance and hardness. This behavior could be shown with these measurements. Compared to the other multilayers without TTIP, the required scratch hardness increased by more than a factor of 2. The addition of TTIP also exceeded the scratch hardnesses of the earlier experiments.

The roughness of specimens has a significant influence on scratch hardness, which is further affected by the introduction of Cu particles into the coating—the value of roughness increases from a R_a_ value below 0.2 µm to approximately 1 to 1.5 µm. A comparison of the “Slightly Acidic One Layer” (sol–gel without particles) with the “Copper Sol–Gel One Layer” and “Zinc Sol–Gel One Layer” highlights the impact of roughness on scratch hardness. While molten Zn particles do not increase roughness, scratch hardness is comparable to the “Slightly Acidic One Layer”. Copper particles, which do not melt strongly, increase roughness and reduce scratch hardness.

**Figure 16:**
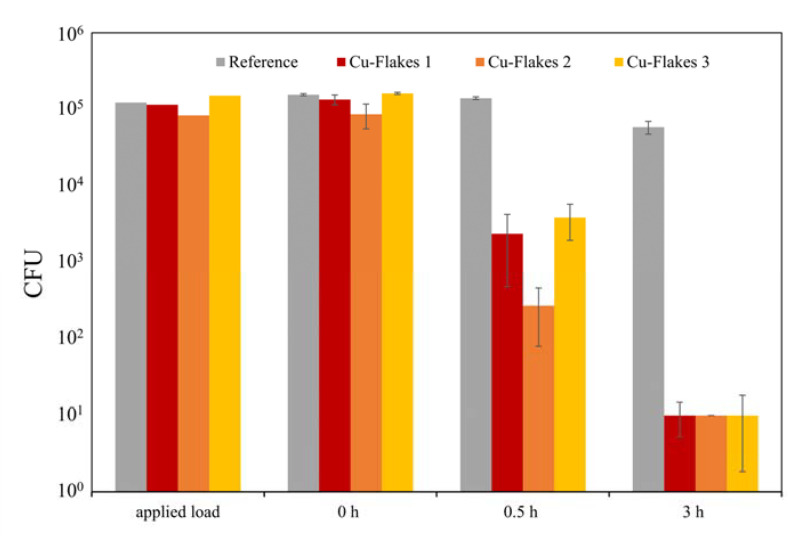
The measurements show the positive effect of multilayers (two and three layers) for friction coefficient reduction. The friction coefficients of the systems remained constant over 10,000 cycles at approx. 1.4 (monolayer) and 0.65 (multilayer).

The tribological outcomes demonstrate the influence of distinct scratch hardness between monolayer and multilayer systems. The multilayer coatings demonstrate a reduction in the coefficient of friction. This is an outcome of the augmented flexibility in the layer system, which is also perceptible in the coating systems employed in this investigation [11,12]. Remarkably, the coefficient of friction diminishes from approximately 1.4 to 0.65 (Figure 16).

#### 2.2.3. Wettability

**Figure 17:**
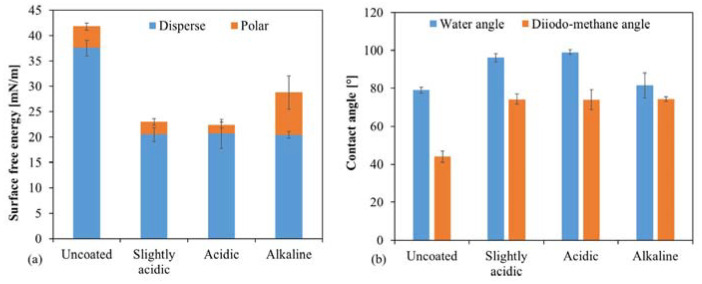
Contact angle measurements with water and diiodomethane: (**a**) shows the surface free energy of the layers, which depends on the pH in the sol–gel system; (**b**) Increasing pH decreased the contact angle of water compared to samples with lower pH value.

Another way to functionalize the surface without applying additional particles or precursors to the layer through the plasma can be realized by varying the catalyst method. The standard sol–gel system has a pH of about 5, in which a contact angle of water on the layer of about 95 to 97° is achieved. If the pH is lowered further by adding an acid (1 M HCl), the contact angle increases slightly (98 to 101°); the polar part of the free surface energy of the layer decreases (Figure 17). This change became even more visible when the sol–gel system was catalyzed not acidic but basic at a pH value of 13–14 by adding a 1 M NaOH. This increase in the pH value caused the system to react within 5 min without the influence of temperature or radiation. The plasma then cross-linked the layer even more. This catalysis leads to a strong increase in the polar part of the surface free energy. Although the coating is still hydrophobic, the contact angle of water drops to approx. 80 to 82°.

The alteration of surface energy has significant implications for biological applications and the creation of self-cleaning layers. By modifying the contact angle and distribution of surface free energy, the wettability of coatings can be controlled to suit various circumstances and objectives. A high pH system can achieve greater wettability, while a low pH system can achieve lower wettability. Furthermore, the sol–gel system’s pH change enables the utilization of other plasma parameters, such as higher current or lower substrate-plasma distance. The internal cross-linking process commences at a high pH without plasma, allowing higher temperatures and radiation to be applied to the layer, thereby accelerating the curing process or applying an adhesion promoter layer directly.

**Figure 18:**
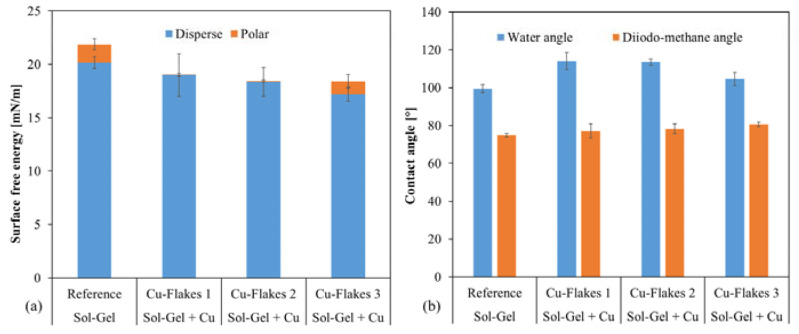
Contact angle measurements with water and diiodomethane: (**a**) shows the surface free energy of the layers with Cu-flakes (Cu-flakes 1 to 3) and a reference without flakes; (**b**) By introducing the flakes into the layer, the water contact angle increased by up to 15°. This decreased again when the loading was too high but remained higher compared to the pure sol–gel layer (reference).

The water contact angle was further increased by introducing Cu flakes into the layer. The contact angle reached a maximum value of approx. 115° (Figure 18). This change can be attributed to the modified surface.

### 2.3. Antibacterial Tests

**Figure 19:**
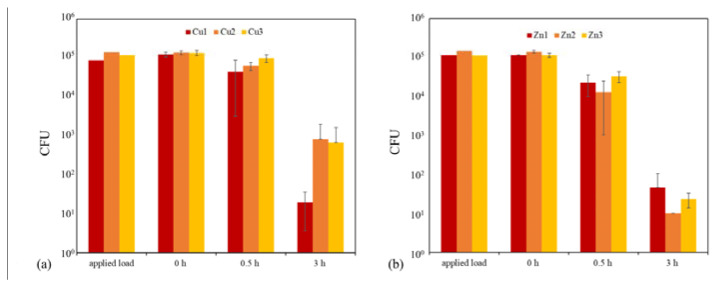
Results of antibacterial tests with *S. aureus* on the substrates coated with sol–gel, and microparticles: (**a**) comparison of the degradation of infectious load on sol–gel coatings with three Cu parameters at three-time points (0, 0.5, and 3 h); (**b**) comparison of the degradation of infectious load on sol–gel coatings with three Zn parameters at three-time points (0, 0.5, and 3 h).

**Figure 20:**
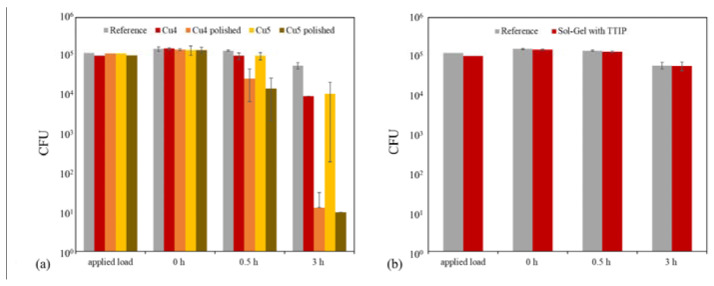
Results of antibacterial tests with *S. aureus* on the substrates coated with sol–gel and microparticles or with TTIP compared to a reference sample: (**a**) comparison of the degradation of the infectious load on sol–gel coatings with two Cu parameters (unpolished or polished) at three time points (0, 0.5, and 3 h); (**b**) comparison of the degradation of the infectious load on sol–gel coatings with TTIP at three time points (0, 0.5, and 3 h).

Veneer-lacquer composites and stainless steel can be equipped with biocidal properties using the sol–gel coating system. This is achieved by applying a Cu or Zn micro powder onto the still-wet sol–gel coating, which is then cured to obtain an effective antimicrobial effect. It is essential to note that a successful antibacterial outcome necessitates a reduction in CFU of approximately 3 log levels, resulting in a 99.9% decrease in the infectious load [23,24]. The antibacterial effect of Cu3 and Zn1–3 samples was good after 3 h due to freely dispersed particles on the surface (Table 8, Figure 19). On the other hand, in the case of samples Cu1 and Cu2, no sufficient antibacterial effect could be detected. Regrettably, the tests caused particles to detach from the layer. However, in the case of samples Cu4 and Cu5, the particles were firmly embedded within the sol–gel coating and fully concealed. In the antibacterial tests, no effect was detected in these samples. Therefore, the samples Cu4 and Cu5 were polished afterward to obtain particles freely on the surface. This post-treatment enabled an antibacterial effect in the subsequent tests (Table 8, Figure 20). The Chwatal et al. [10] paper describes this behavior in even more detail.

No antibacterial effect was detected in the samples with the Sol–Gel TTIP coating. The coatings behaved the same way as the pure sol–gel coatings without functionalization (Table 8, Figure 20).

Tests were conducted on sol–gel coatings both with and without Cu addition. The findings revealed that Cu particles were essential in achieving the intended outcome. Surfaces lacking Cu particles showed a reduction of only 10% for *Staphylococcus aureus* (*S. aureus*) after 30 min and approximately 62% after three hours. This decline may be attributed to a lack of nutrients and fluids or the rough surface’s mechanical effect, which can result in the bacterium’s irreversible destruction.

**Figure 21:**
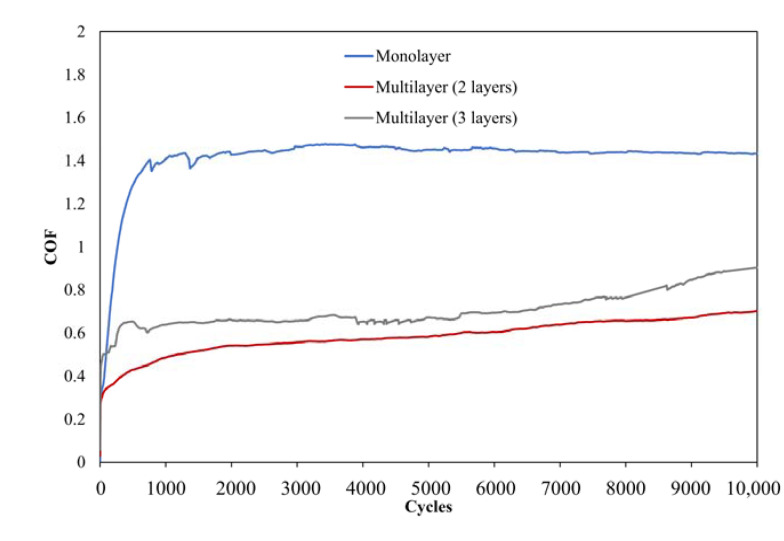
Results of antibacterial tests with *S. aureus* on the substrates coated with sol–gel, and Cu-Flakes: comparison of the degradation of infectious load on sol–gel coatings with three Cu-Flakes parameters at three-time points (0, 0.5, and 3 h) and a reference measurement (pure sol–gel layer without flakes).

Flat Cu flakes with a size of 34 µm were used instead of the “spherical” Cu particles to accelerate the antibacterial effect further. Due to the elongated and flat shape, the flakes lay better on the sol–gel surface and did not sink into the layer as fast as the “spherical” Cu particles. The flakes also formed “stacks”. As a result, more flakes lie on the surface, increasing the Cu concentration. In addition, the plasma parameters were also adjusted. Only the powder carrier gas flow was used during the flakes’ application, and the plasma acted as a nozzle. During this process, the plasma was not activated. As a result of these two adjustments, a higher Cu concentration formed on the surface. This led to faster degradation of the bacterial load. After 0.5 h, a degradation of over 98% was achieved for almost all three parameters, which is necessary to speak of an antibacterial effect (Figure 21). In comparison, the samples with the “spherical” particles after 0.5 h without post-treatment were between 25 and 65% and with post-treatment below 90% (Table 8).

## 3. Conclusions

This study showcased the use of an atmospheric pressure plasma jet system to enhance the application, functionalization, and curing process of a thermal and UV-curing sol–gel system. The plasma enables fast curing, while the UV radiation and heat accelerate the curing of thick sol–gel layers more efficiently than current methods. The degree of cross-linking and chemical reactions over time can be determined through FTIR spectra analysis. The Si-O-Si bond effectively describes the built Si network and the addition of TTIP to the sol–gel mixture increases scratch resistance and hydrophobicity. Plasma allows for precise and quick modification of layer properties, including the introduction of particles or flakes to enhance antibacterial properties. pH changes can also be used to vary surface free energy and hardness for a wider range of applications. With the option to utilize a highly scratch-resistant monolayer or multifunctional multilayer sol–gel system, layer thicknesses can range from 10 to 250 µm on various substrates. A difference in scratch hardness between monolayer and multilayer systems was also demonstrated. This also results in friction values that differ greatly from one another. This process can reduce the infectious load by 99.99% within 3 h (after 30 min 90%). It can even be accelerated by using Cu-flakes instead of Cu or Zn particles, achieving a reduction of 98%-99% in 30 min and of 99.99% within 3 h.

## 4. Experimental Setup and Characterization

### 4.1. Sol–Gel Preparation

The dual-cure hybrid sol–gel was prepared by combining 1.9 g of 3-(trimethoxysilyl)propyl methacrylate (MERCK CHEMICALS AND LIFE SCIENCE GESMBH Life Science, Vienna, Austria, 2.2 g of Ebecryl^®^ 8890 (Allnex, Werndorf, Austria), and 0.8 g of methanol (MERCK) in a resin beaker (resin). The resulting mixture was stirred for 5 min before introducing a photoinitiator mixture (radical starter) of 1-Hydroxycyclohexyl phenyl ketone and Benzophenone (BASF, Eugendorf, Austria). In the third step, 4.3 g of (3-aminopropyl)triethoxysilane, abbreviated APTES, (hardener) (MERCK) and 0.5 g of a non-stick agent were added, and the resulting mixture was stirred for an additional 5 min. The dual-cure hybrid sol–gel was then ready for coating and utilization [3,4,17,25,26,27,28].

**Figure 22:**
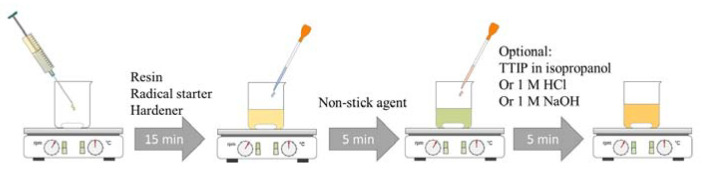
Multi-step sol–gel production with modifications in the last step.

In addition, a solution of titanium tetraisopropylate (TTIP) in isopropanol (20% TTIP) was added to obtain a functionalization of the sol–gel. Therefore, 1 g of the TTIP/isopropanol solution was added to 4 g of the sol–gel. The aim was to add higher scratch resistance to the sol–gel and convert the TTIP into TiO_2_ by the plasma, thus obtaining a self-cleaning effect. Antibacterial tests should confirm this effect (Figure 22).

Furthermore, the influence of pH value on the curing behavior and the resulting layer properties was investigated. For pH values over 10, 1.8 g of a 1 M NaOH solution was added to 9 g sol–gel, and for pH values below 5, 1.8 g of a 1 M HCl solution.

### 4.2. Sample Preparation

The study used wood veneer (F. LIST, Thomasberg, Austria) and stainless steel 1.4301 as substrates. The veneer was composed of a base plate, a honeycomb core, and the wood veneer as a top plate. Before the first treatment with sol–gel, the veneer was impregnated with varnish from the company F.LIST. This structure and the stainless-steel substrate were then functionalized with a sol–gel layer at Joanneum Research.

Subsequently, a wet sol–gel layer of 20 µm was applied with a manual film applicator (BYK-Chemie GmbH, Wesel, Germany) (Table 9).

The substrates were mounted and moved on an XY Linear robot for plasma treatment.

### 4.3. Curing Process with Atmospheric Pressure Plasma

**Figure 23:**
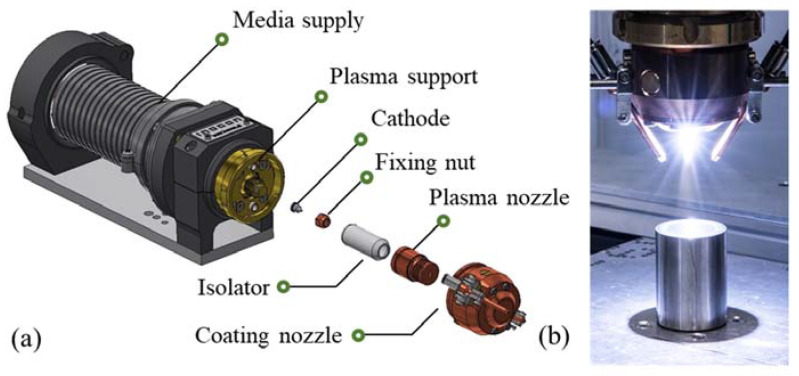
(**a**) Systematic design of the atmospheric pressure plasma nozzle; (**b**) Plasma nozzle in operation.

INOCON Technologie GmbH’s atmospheric pressure plasma jet, InoCoat 3^®^, was utilized to achieve the curing and functionalization of sol–gel layers through the combination of thermal and radiation energy, including UV radiation (Figure 23, Table 2, Table 3 and Table 6).

Plasma was activated to harden the sol–gel and achieve functionalization using specific precursors and particles [23,24,29,30]. Due to the high thermal energy of the plasma, the powder burst and underwent a slight melting on its surface. The particles were shot at high speed onto the still liquid sol–gel coating. The timing at which the particles were applied to the sol–gel varied. First, the particles (plasmECK Cu 1001 (10 µm diameter) and VP68334/G Zn (10 µm diameter), ECKART, Hartenstein, Germany) or flakes (VP72166/G (34 µm diameter), ECKART) were applied to the still untreated sol–gel layer. In another approach, the particles were applied to a sol–gel briefly treated with the plasma. This variation was to show the change in the penetration depth of the particles into the layer.

The precursors, e.g., HMDSO, were adhesion promoters between the individual sol–gel layers [5,18,31]. Through the plasma process, HMDSO reacts to form a silicon or SiO_2_ layer. This layer can be applied hydrophilic or hydrophobic to the substrate by varying the plasma parameters. The hydrophilic layer was used as an adhesion promoter layer in these experiments.

After the HMDSO layer was applied to the cured sol–gel layer by plasma, the next sol–gel layer could be applied. This procedure was repeated four times in this work, resulting in a five-layer sol–gel system (Figure 11).

### 4.4. Post-Growth Characterization

#### 4.4.1. Optical Characterization

Fourier transform infrared spectroscopy (FTIR) was utilized as the characterization method to assess the curing process and its parameters’ effects on the sol–gel. This approach enables quick quality assessment of the curing process, making it an ideal choice. Since the sol–gel system is silane-based, it is expected to exhibit peaks within the 1200 cm^−1^ to 800 cm^−1^ range. The measurements were conducted with a Shimadzu IRSpirit Fourier transform infrared spectrophotometer using damped total internal reflection mode (Shimadzu, Austria). The Shimadzu LabSolutions IR software was used to analyze the spectra after performing baseline correction and smoothing for each spectrum. Additionally, all spectra were normalized to their highest intensity peaks, 1067 cm^−1^ for the sol–gel spectrum after 0 h and 1013 cm^−1^ for the sol–gel spectrum after 24 h. The LabSolutions software was utilized to analyze the “stacked” spectra for display.

#### 4.4.2. Structural Characterization

Various techniques were utilized to examine the characteristics of the sample. The TESCAN VEGA3 microscope equipped with a heated tungsten filament and a 20 kV high-voltage power supply was used to capture SEM secondary electron and backscattered electron images. EDXS with an Oxford detector was utilized to perform chemical analyses at 10–20 kV and a probe current of 0.6–1.3 µA with a spot size of approximately 500 nm. Results were normalized to 100%.

According to DIN EN ISO 19403, contact angle measurement is a reliable method to assess a solid’s wetting behavior. This involves measuring the contact angle of a test liquid (water and diiodomethane) at the three-phase point and calculating the surface free energy of the coating. The DROP SHAPE ANALYZER-DSA30 from Kruess was used to carry out these measurements.

The Zeiss Axio Scope.A1 LED microscope was utilized for the purpose of carrying out the microscopy. Images were captured and processed using Zeiss’s ZEN 2 core v2.4 software.

#### 4.4.3. Tribological Characterization

For the precise measurement of scratch hardness, we utilized the TQC Sheen Hardness Test Pro as per the ISO 1518-2 standard. To ensure accuracy, we used a specialized 1.0 mm diameter tip.

To ensure accurate measurement of coating thicknesses, we utilized the Veeco Dektak 150 Surface Profiler and its accompanying software, along with Microsoft Excel 2016. First, a 5 mm segment of the substrate was covered with Kapton adhesive tape before applying the coating. After curing, the segment was removed, creating a step that allowed for precise measurement of the coating thickness. To guarantee precision, we conducted three measurements of each sample and calculated standard deviation using Excel.

The tribology tests were performed with Anton Paar’s ball-on-disc tribometer TRB3 with a load of 1 N. A polyamide ball with a diameter of 6 mm was used as a static counter body for this purpose. The sliding distance was 630 m (10,000 cycles).

#### 4.4.4. Biological Characterization

In order to conduct a thorough evaluation of the antibacterial properties of different surfaces, a meticulous test was carried out according to ISO 22196 guidelines, albeit with modifications. The ISO 22196 strain was cultured overnight on Columbia Blood agar plates (BD, Heidelberg, Germany) at a temperature of 36 °C ± 2 °C. Subsequently, cell material was obtained from these plates and diluted in a 1:500 dilution of tryptic soy broth (Oxoid, Wesel, Germany) in distilled water to create a stable environment for the bacteria, without promoting their growth. Bacterial solutions with a density of 1 × 10^8^ colony-forming units (CFU)/mL were achieved through the use of a VITEK^®^ DensiCHEK instrument (Biomerièux, Vienna, Austria). To ensure precise and reliable outcomes, a target concentration of 2.5–10 × 10^5^ cells/mL was pipetted onto the test surfaces, and a 4 × 4 cm polyethylenterephthalat (PET) film (VWR International, Vienna, Austria), sterilized beforehand, was used to evenly distribute the suspension. The inoculated test specimens were incubated in a wet chamber at 36 °C ± 2 °C with approximately 96% relative humidity (RH) for 0.5 h and 3 h. The number of bacterial cells immediately following inoculation (0 h) on both untreated and treated samples was determined to ensure an appropriate initial concentration on each surface tested. The surviving bacterial cells were collected using a neutralizer, and suspensions were diluted with 1× phosphate buffered saline (Carl Roth GmbH + Co. Kg, Karlsruhe, Germany). The diluted suspensions were applied in duplicate to tryptic soy agar (TSA, VWR International LTD., Austria), and CFUs were counted after incubation for 24 h at 36 °C ± 2 °C. A detection limit of 10 CFU was established if no colonies could be observed on the plates since 10 mL of the neutralization medium was used. These precise procedures enabled a confident assessment of the antibacterial activity of the surfaces, leading to accurate and reliable results.

## Figures and Tables

**Table 1:** Assignment of the FTIR peaks.

Assignments	Peak (cm^−1^)	Relative Intensity * Slightly Acidic|Alkaline 0 h, 24 h|0 h, 24 h	References
Si-OH	950	0.35, 0.29|0.34, 0.24	[3,4,13,14,15,16]
Si-OCH_2_CH_3_	1163	0.33, 0.26|0.34, 0.22	[3,4,17,18]
	1100	0.68, 0.62|0.74, 0.65	
	1067	0.94, 0.63|0.97, 0.60	
	950	0.35, 0.29|0.34, 0.24	
Si-O-Si	1039	1.00, 0.83|1.00, 0.80	[3,4,13,14,15,16,17,18]
	1013	0.85, 1.00|0.84, 1.00	
	700	0.23, 0.25|0.21, 0.25	
Si-CH_3_	1260	0.14, 0.10|0.39, 0.10	[3,4]
	800–760	0.48, 0.31|0.48, 0.30	
C=C	1640	0.11, 0.14|0.11, 0.16	
C=O	1716	0.29, 0.30|0.29, 0.29	[3,4,5]

* The relative peak intensities of the spectra shown in Figure 1 have been provided, with each spectrum normalized to its highest peak (1039 cm^−1^ for cured samples after 0 h and 1013 cm^−1^ for cured samples after 24 h). The order in which the intensities are presented is as follows: Slightly Acidic 0 h, Slightly Acidic 24 h, Alkaline 0 h, and Alkaline 24 h.

**Table 2:** To treat the sol–gel coated sample with powder via plasma, use the following parameters: current (C), moving speed (MS), treatment time (TT), plasma gas (PG), gas flow (GF), substrate-plasma distance (S-P D), and powder flow (PF).

Plasma Powder Treatment								
Parameter	Powder	C [A]	MS [mm/s]	TT [s]	PG	GF [L/min]	S-P D [mm]	PF [g/min]
Powder 1	Copper	180	500	10	Argon	10	150	2
Powder 2	Copper	120	500	10	Argon	10	150	2
Powder 3	Zinc	180	500	10	Argon	10	150	2
Powder 4	Zinc	120	500	10	Argon	10	150	2
Powder 5	Copper Flakes	-	50	100	-	-	250	2
Powder 6	Copper Flakes	-	75	75	-	-	250	2
Powder 7	Copper Flakes	-	100	50	-	-	250	2

**Table 3:** To cure the sol–gel coated sample through plasma, the following parameters were utilized: current (C), moving speed (MS), treatment time (TT), plasma gas (PG), gas flow (GF), and substrate-plasma distance (S-P D).

Plasma Treatment						
Parameter	C [A]	MS [mm/s]	TT [s]	PG	GF [L/min]	S-P D [mm]
Plasma 1	250	100	160	Argon	10	250
Plasma 2	180 250	100	40 120	Argon	10	250
Plasma 3	180 250	100	80 80	Argon	10	250
Plasma 4	180 250	100	40 80	Argon	10	250

**Table 4:** Silicon, oxygen, and titanium distribution in the sol–gel and sol–gel top layers.

	Without TiO_2_ − Top Layer		With TiO_2_ − Top Layer	
Element	In Layer	Top Layer	In Layer	Top Layer
O	65.1	65.8	62.7	75.1
Si	32.7	32.0	34.7	5.9
Ti	2.2	2.3	2.6	19.0

**Table 5:** Layer thicknesses of two multilayer sol–gel systems with and without mixed-in powder were measured with the “Dektak 150 Surface Profiler, Veeco, Plainview, New York, USA”.

Coating	Layer Thickness [µm]
Sol–Gel with TTIP	145.8 ± 9.7
Sol–Gel with TTIP + TiO_2_	232.5 ± 25.3

**Table 6:** Specific parameters were utilized to treat the sol–gel coated sample with a precursor through plasma. These parameters included current (C), moving speed (MS), treatment time (TT), plasma gas (PG), gas flow (GF), substrate-plasma distance (S-P D), and precursor flow (PrF).

Plasma Adhesion Layer Treatment								
Parameter	Precursor	C [A]	MS [mm/s]	TT [s]	PG	GF [L/min]	S-P D [mm]	PrF [mL/min]
Adhesion 1	HMDSO	180	100	40	Argon	10	250	80

**Table 7:** Scratch test measurements on various sol–gel coatings with different film thicknesses. Three measurements per coating were performed.

Coating	Parameters	Scratch Hardness [N]
Alkaline One Layer	Plasma 1	12.7 ± 0.5
Slightly acidic One Layer	Plasma 1	14.3 ± 0.5
Acidic One Layer	Plasma 1	14.7 ± 0.5
Alkaline Three Layers	Plasma 1	8.3 ± 0.5
Slightly acidic Three Layers	Plasma 1	8.7 ± 0.5
Acidic Three Layers	Plasma 1	6.0 ± 0.8
Copper Sol–Gel One Layer	Plasma 1	12.7 ± 0.5
Zinc Sol–Gel One Layer	Plasma 1	14.7 ± 0.5
Sol–Gel with TTIP Five Layers	Plasma 2	18.0 ± 0.8
Sol–Gel with TTIP + TiO_2_ Five Layers	Plasma 2	17.7 ± 0.5

**Table 8:** Summary of the different sample designations with the corresponding coating and curing parameters and the antibacterial effect on *S. aureus* after 0.5 and 3 h.

Sample	Parameter	Reduction in CFU after 0.5 h	Reduction in CFU after 3 h
Cu1	Powder 1 Plasma 1	63.53%	99.98%
Cu2	Powder 2 Plasma 1	54.30%	99.39%
Cu3	Powder 1 Plasma 2	26.72%	99.50%
Zn1	Powder 3 Plasma 1	80.26%	99.96%
Zn2	Powder 4 Plasma 1	90.87%	99.99%
Zn3	Powder 3 Plasma 2	71.20%	99.98%
Cu4	Powder 1 Plasma 4	34.82%	93.89%
Cu4 polished	Powder 1 Plasma 4	81.70%	99.99%
Cu5	Powder 2 Plasma 4	29.22%	92.49%
Cu5 polished	Powder 2 Plasma 4	89.70%	99.99%
Cu-Flakes 1	Powder 5 Plasma 2	98.23%	99.99%
Cu-Flakes 2	Powder 6 Plasma 2	99.68%	99.99%
Cu-Flakes 3	Powder 7 Plasma 2	97.60%	99.99%
Sol–Gel with TTIP	Plasma 2	11.86%	62.06%
Reference	Plasma 2	9.94%	62.42%

**Table 9:** Sol–Gel Coating parameters and conditions.

Sol–Gel Coating				
Sample	Layer Thickness Applied [µm]	Layer Thickness after Treatment [µm]	Room Temperature [°C]	Humidity %
Sol–Gel Coating (any pH value)	20	5	25	60
TTIP Sol–Gel Coating	20	5	25	60

## Data Availability

The data used in this study are available from the corresponding author (simon.chwatal@joanneum.at) upon reasonable request.
